# Priority-setting to integrate sexual and reproductive health into universal health coverage: the case of Malaysia

**DOI:** 10.1080/26410397.2020.1842153

**Published:** 2020-11-25

**Authors:** Shiang Cheng Lim, Yee Chern Yap, Sima Barmania, Veloshnee Govender, Georges Danhoundo, Michelle Remme

**Affiliations:** a Country Technical Lead, Better Health Programme Malaysia, RTI International Malaysia, Kuala Lumpur, Malaysia/Post-doctoral Fellow, International Institute for Global Health, United Nations University, Kuala Lumpur, Malaysia.; b Research Intern, International Institute for Global Health, United Nations University, Kuala Lumpur, Malaysia; c Consultant, International Institute for Global Health, United Nations University, Kuala Lumpur, Malaysia; d Scientist, Department of Sexual and Reproductive Health and Research (SRH), World Health Organization, Geneva, Switzerland; e Research Lead, International Institute for Global Health, United Nations University, Kuala Lumpur, Malaysia

**Keywords:** priority-setting, sexual and reproductive health, universal health coverage, context–mechanism–outcome, pregnancy, safe delivery and post-natal care, gender-based violence (GBV), abortion

## Abstract

Despite increasing calls to integrate and prioritise sexual and reproductive health (SRH) services in universal health coverage (UHC) processes, several SRH services have remained a low priority in countries’ UHC plans. This study aims to understand the priority-setting process of SRH interventions in the context of UHC, drawing on the Malaysian experience. A realist evaluation framework was adopted to examine the priority-setting process for three SRH tracer interventions: pregnancy, safe delivery and post-natal care; gender-based violence (GBV) services; and abortion-related services. The study used a qualitative multi-method design, including a literature and document review, and 20 in-depth key informant interviews, to explore the context–mechanism–outcome configurations that influenced and explained the priority-setting process. Four key advocacy strategies were identified for the effective prioritisation of SRH services, namely: (1) generating public demand and social support, (2) linking SRH issues with public agendas or international commitments, (3) engaging champions that are internal and external to the public health sector, and (4) reframing SRH issues as public health issues. While these strategies successfully triggered mechanisms, such as mutual understanding and increased buy-in of policymakers to prioritise SRH services, the level and extent of prioritisation was affected by both inner and outer contextual factors, in particular the socio-cultural and political context. Priority-setting is a political decision-making process that reflects societal values and norms. Efforts to integrate SRH services in UHC processes need both to make technical arguments and to find strategies to overcome barriers related to societal values (including certain socio-cultural and religious norms). This is particularly important for sensitive SRH services, like GBV and safe abortion, and for certain populations.

## Résumé

En dépit d’appels croissants invitant à intégrer et prioriser les services de santé sexuelle et reproductive (SSR) dans les procédures de la couverture santé universelle (CSU), plusieurs services de SSR continuent de recevoir une faible priorité dans les plans nationaux de la CSU. Cette étude vise à comprendre le processus de définition des priorités des interventions de SSR dans le contexte de la CSU, en s’inspirant de l’expérience en Malaisie. Un cadre d’évaluation réaliste a été adopté pour examiner le processus de définition des priorités pour trois interventions repères de SSR : soins pendant la grossesse, l’accouchement sûr et après la naissance; services en matière de violence sexuelle; et services liés à l’avortement. L’étude a utilisé une conception qualitative à plusieurs méthodes, notamment un examen des publications et documents de même que 20 entretiens avec des informateurs clés, pour étudier les configurations contexte-mécanisme-résultat qui ont influencé et expliqué le processus de définition des priorités. Quatre principales stratégies de plaidoyer ont été identifiées pour la priorisation des services de SSR, à savoir : 1) créer une demande publique et un soutien social, 2) lier les questions de SSR avec les programmes publics ou les engagements internationaux, 3) recruter des champions à l’intérieur et l’extérieur du secteur de la santé publique, et 4) recadrer les questions de SSR comme thèmes de santé publique. Si ces stratégies ont réussi à déclencher des mécanismes, comme une compréhension mutuelle et une volonté accrue des décideurs d’accorder la priorité aux services de SSR, le niveau et l’étendue de la priorisation étaient influencés par des facteurs contextuels internes et externes, en particulier le contexte socio-culturel et politique. La définition des priorités est une procédure de prise de décision politique qui reflète les valeurs et normes de la société. Les activités pour intégrer les services de SSR dans les processus de la CSU doivent présenter des arguments techniques et trouver des stratégies pour surmonter les obstacles relatifs aux valeurs sociétales (y compris certaines normes socioculturelles et religieuses). C’est particulièrement important pour les services de SSR délicats, comme ceux qui concernent la violence sexuelle et l’avortement, ou sont destinés à certaines populations.

## Resumen

A pesar del creciente número de llamadas para integrar y priorizar los servicios de salud sexual y reproductiva (SSR) en los procesos de cobertura universal de salud (CUS), varios servicios de SSR continúan siendo una baja prioridad en los planes de CUS de los países. Este estudio busca entender el proceso de establecer prioridades con relación a las intervenciones de SSR en el contexto de la CUS, basándose en la experiencia de Malasia. Se adoptó un marco de evaluación realista para examinar el proceso de establecer prioridades para tres intervenciones de seguimiento de SSR: servicios de atención al embarazo, partos seguros y atención posnatal; servicios de atención a violencia de género (VG); y servicios relacionados con el aborto. El estudio utilizó un diseño multimétodo cualitativo, que incluyó una revisión de la literatura y documentación, y 20 entrevistas a profundidad con informantes clave, con el fin de explorar las configuraciones de contexto–mecanismo–resultado que afectaron y explicaron el proceso de establecer prioridades. Se identificaron cuatro estrategias clave de promoción y defensa para la priorización eficaz de los servicios de SSR: (1) generar demanda pública y apoyo social, (2) vincular los asuntos de SSR con agendas públicas o con compromisos internacionales, (3) incluir a defensores del sector de salud pública, tanto a nivel interno como externo, y (4) replantear los asuntos de SSR como asuntos de salud pública. Aunque estas estrategias lograron activar mecanismos, tales como comprensión mutua y mayor aceptación de los formuladores de políticas para priorizar los servicios de SSR, el nivel y grado de priorización se vieron afectados por factores contextuales tanto internos como externos, en particular el contexto sociocultural y político. El establecimiento de prioridades es un proceso de toma de decisiones políticas que refleja los valores y las normas sociales. Los esfuerzos por integrar los servicios de SSR en los procesos de CUS deben presentar argumentos técnicos y encontrar, estrategias para superar las barreras relacionadas con los valores sociales (incluidas ciertas normas socioculturales y religiosas). Esto es de particular importancia para los servicios de SSR sensibles, como los servicios de VG y aborto seguro, y para ciertas poblaciones.

## 1. Background/Introduction

Both Universal Health Coverage (UHC) and universal access to Sexual and Reproductive Health (SRH) services are closely linked and mutually-reinforcing targets under the Sustainable Development Goal (SDG) on healthy living.^1^ UHC is the aspiration to ensure that “All people obtain the health services they need – prevention, promotion, treatment, rehabilitation and palliation – without risk of financial ruin or impoverishment, now and in the future”.^2^ To progress towards UHC, countries are urged to strengthen their health systems, and to progressively increase financial protection and equitable access to the full spectrum of quality health services needed across the lifespan by all people, including the most vulnerable and marginalised.^3^ Countries with more significant resource constraints are recommended to start with the prioritisation of essential health services and reaching those with the least access, as they work towards UHC.^4^

There have been increasing calls to integrate comprehensive SRH services* over the life course into the essential packages of health services (EPHS) or health benefits packages (HBPs) in national UHC plans.^1,5,6^ While maternal health services, including obstetric emergencies, and family planning are typically included in these packages, others such as safe abortion, gender-based violence (GBV), cancer screening and fertility care have been given less attention and are mainly financed through out-of-pocket expenditure (OOPE) or external donor funding.^7–10^ This raises serious concerns for effective coverage, equity and financial protection. Globally, almost all 4.3 billion people of reproductive age will lack access to adequate SRH services at some point in their lifetime.^11^ This is particularly pronounced for certain groups that tend to be excluded from services, such as adolescents, unmarried women, migrants, people living with HIV, people with disabilities, and lesbian, gay, bisexual, transgender, queer, and intersex (LGBTQI) people.^11^ Low-income households also bear a heavier financial burden when seeking SRH services, compared to higher-income households,^12^ and a higher incidence of catastrophic expenditures.^7^

Universal access to SRH services will require increased government prioritisation and public financing, as part of a clearly defined pathway towards UHC that addresses people's unique SRH needs.^1,9^ SRH issues need to become political priorities, and their solutions need to be adequately resourced by the health system. This prioritisation process is critical to ensure that quality and cost-effective SRH interventions are made available, within the resource envelope, and are accessible and acceptable to vulnerable, marginalised and hard-to-reach populations.^1,3^ Although a strong case had been made for prioritising SRH interventions and services in UHC, this is often constrained by the cultural and political sensitivities related to sexuality, reproductive choices, and gender inequality, resulting in policy, regulatory and legislative barriers to providing and accessing SRH services.^11^ There is limited evidence on why certain SRH interventions or services are being prioritised in UHC plans, what the most effective strategies have been to enable this prioritisation, and what it takes for this to happen in specific contexts.^7,13^

### 1.1. Malaysia as a case study

Since its independence in 1957, Malaysia has made remarkable progress in extending health service coverage, and improving health outcomes, particularly for maternal and child health.^14^ The maternal mortality rate (MMR) fell substantially, from 280 per 100,000 live births in 1957 to 44 in 1991 and 25 in 2018.^14–16^ Malaysia's population accesses healthcare through a two-tier system: a subsidised and tax-funded public healthcare system; and a private healthcare system, predominantly financed through OOPE.^17^ The Ministry of Health's (MOH) facilities provide free (or almost free) healthcare services for the majority of Malaysians, including SRH services across the spectrum of prevention to treatment.^18^ The 2018 UHC Index showed that Malaysia has relatively high coverage of essential services and a low risk of financial hardship, compared to neighbouring countries.^19^ In addition, Malaysia was a pioneer in rolling out the One Stop Crisis Centre model for survivors of GBV in 1994.^20^

Pregnancy, childbirth and maternal care have been prioritised as part of the country's essential services and fully integrated at different levels within the public health system. However, other SRH services have received less attention and often remain taboo subjects, due to the restrictive interpretations of cultural and religious norms and practices that result in stigma and embarrassment, in combination with policy, regulatory and legislative barriers.^21^ These barriers have further impeded the rights and access to SRH services by certain groups, including unmarried women, adolescents and young people, migrants, refugees and asylum seekers, indigenous communities, LGBTQI persons, people living with HIV and persons with psychosocial and developmental disabilities.^21,22^

As such, the objective of this study is to draw on the Malaysian experience to understand the priority-setting process of SRH in UHC, including what needs to happen, through what mechanisms, in light of what contextual factors. It also aims to understand how critical choices and decisions have led to the different level of prioritisation of different types of SRH services within the national UHC trajectory in Malaysia. The purpose of this case study is to provide insights into the processes and pathways that have the potential to help countries to advance towards universal access to SRH services.

## 2. Methods

In this paper, the priority-setting process is defined as *“to select among different options for addressing the most important health needs … given limited resources (rationing)”.*^3^ As such, priority-setting is inherently a political process that reflects population demand and needs, as well as the societal values and trade-offs between various criteria and considerations.^3^ It occurs at all levels of a health system, from macro to meso and micro level, through various mechanisms.^3,23–25^ The criteria identified by WHO for setting priorities relate to both prioritising health problems or issues, and prioritising solutions or interventions to address those problems. While disease burden is central for the former, considerations around effectiveness, cost, acceptability and fairness are key for the latter.^3^

The priority-setting process for SRH services is even more challenging, given that many SRH issues and interventions are subject to legal restrictions (e.g. abortion laws, criminalisation of consensual sex among adolescents) and cultural norms related to gender, homosexuality, sex work and age of consent, preventing unmarried women, adolescents, sex workers and LGBTQI persons from accessing services.^11^

While comprehensive SRH services are clearly defined, we focus on a sub-set of tracer interventions to explore the strategies, mechanisms, stakeholders and other contextual factors in Malaysia that influenced the prioritisation and integration of SRH services, mainly at the macro or policy level. Three tracer interventions were selected to capture lessons from different types of SRH interventions, based on how established and controversial they are, namely:

- **Established interventions:** Pregnancy and safe delivery care, which has been prioritised and included in EPHS or HBP by most countries;
- **Newly emerging interventions:** Gender-based violence services, which have been increasingly recognised as a complex public health issue with legal, social, cultural, economic and psychological dimensions that need to be prioritised in the context of UHC;
- **Sensitive and controversial interventions:** Safe abortion services and post-abortion care, which have been systematically neglected in the context of UHC, due to restrictive laws and policies influenced by social norms and stigma towards abortion.

### 2.1. Conceptual framework

This study adopted a realist evaluation framework to identify not only the strategies that worked to integrate SRH services in UHC, but also how these strategies worked, for whom, and in what circumstances.^26^ This framework is built on four key concepts to explain the impact of a strategy, programme or intervention: context (C), mechanisms (M), outcomes (O), and their so-called C–M–O configurations. According to Pawson and Tilley,^26^ a mechanism (M) describes the reaction of the actors to the resources provided by the strategy or intervention that may lead it to have a particular outcome. Context (C) is defined as the required conditions for an intervention to trigger those mechanisms to produce particular outcome patterns. This captures both the “for whom” and “in what circumstances” an intervention works. Outcome (O) refers to the practical effects produced by a causal mechanism triggered in a particular context. As such, Pawson and Tilley^26^ argued that whether a strategy or intervention can generate its anticipated outcomes depends on how the underlying generative mechanisms respond in the context within which it is being implemented.

For this analysis, the outcome (O) was defined as the level of prioritisation – high, medium, low – of an SRH intervention in the public UHC plan. An SRH intervention that was both considered to address a priority population health issue, and was allocated significant government resources, was defined as having achieved a “high” level of prioritisation. On the other hand, an intervention that was neither considered a priority issue, nor a priority budget item, had a “low” prioritisation level. Finally, an SRH issue that was viewed as a partial health priority, and only allocated a minimal budget, had a “medium” level of prioritisation.

We identified the priority-setting strategies or specific actions taken by stakeholders in Malaysia to promote the integration of these tracer interventions in the health system. We then defined and examined the mechanisms that enabled and triggered the respective outcome (O) for each SRH tracer. The mechanism was interpreted as stakeholders’ reactions, behaviours or decisions in response to the implemented strategies.

For the analysis of the context (C) in which the mechanism took place, we considered both the outer and the inner contexts in Malaysia. The outer context was defined as the external environment including the service and policy environment, while the inner context focused on the characteristics within an organisation such as organisational structures, resources, staffing, etc.^27^

### 2.2. Data collection

We adopted a qualitative multi-method approach to understand the priority-setting and integration of the three tracer interventions in the national UHC processes, which included a literature and document review and key informant interviews. The approach was expected to increase the credibility of the study by exploring and validating diverse perspectives through a triangulation of information from different sources^28^ to develop a complete understanding of this complex of issue.

#### 2.2.1. Literature search and document review

The literature search was conducted through an iterative process. First, print and electronic official documents, and grey literature published since 1980, were collected and reviewed to get an overview of the issues and gaps related to SRH services in Malaysia, in terms of availability, accessibility, acceptability, quality and financial protection. These documents included research publications, national data, population surveys and census reports, government reports, Ministry of Health policies, strategic plans and reports on health services and health system resources (e.g. human resources, health infrastructure and financing), and reports or independent studies from development partners and multilateral agencies. The feedback and additional documents recommended or provided by key informants complemented the literature search.

#### 2.2.2. Key informant interviews

A stakeholder mapping was conducted based on the initial literature review to identify key informants to be interviewed to fill specific gaps. A total of 11 key informants were selected purposively and an additional nine key informants were introduced through snowballing, based on their expertise, organisation (position and level of seniority) or personal involvement in policy-making or programming for the three SRH tracer interventions, and in particular, their involvement in the priority-setting process over the last 20–30 years.

They were from the government sector (past and present), as well as civil society and academia (Table 1).

**Table 1.** Key informants

Key informants	Number
NGO representatives and UN implementing agencies Sexual and reproductive health (SRH) organisation Women’s organisation LGBTQI organisation Health professional organisation UNHCR (United Nations High Commissioner for Refugees)	7
Academics with medical/MOH background or expertise in UHC and SRH	2
Current health policy-makers Family Health and Development Division (FHDD), MOH Obstetrics and Gynaecology Development Committee (JKPPOG), MOH Malaysian Health Technology Assessment Section (MaHTAS), MOH	3
Former health policy-makers	4
Health provider/medical practitioner	1
Religious leaders	2
Insurance provider	1

Semi-structured interviews were conducted with 20 key informants. The semi-structured topic guides covered the following topics:

- Overview of the state and trends of access to SRH services and UHC in the country
- What were the individual/organisation's role and their participation in priority-setting for the inclusion of either of the three SRH tracer interventions in UHC processes? And what were the outcomes/level of prioritisation of these interventions?
- What were the strategies/measures taken in the country to prioritise these three SRH tracer interventions in the UHC plan and processes?
- What were the enablers and challenges? What worked and what did not work? How did certain strategies work? What contextual factors enabled or impeded them to work?

All interviews were conducted in English. Two interviews were conducted virtually as the key informants were unable to meet face-to-face. Each interview took between 60 and 90 min and written consent was obtained before the interview. All key informants agreed for the interviews to be audio-recorded.

The key informants could only be identified by a sequentially generated ID-number and their stakeholder “type” during data collection, follow-up, data processing, and analysis to ensure confidentiality. All recorded interviews were transcribed by the research team.

Ethical clearance for this study was obtained from the Monash University Human Research Ethics Committee, which is a local university/institution (22718).

### 2.3. Data analysis

A thematic analysis was conducted based on the realist evaluation CMO concepts. The transcripts were coded, codes extracted, and analysed according to recurrent themes using the CMO framework. The themes for the strategies, context, and mechanisms were further organised into sub-categories that could be grouped. The data were also triangulated between different data sources, namely the literature review and key informant interviews, to ensure the validity of the findings as they emerged. Investigator triangulation was also conducted within the research team to discuss and gain multiple perspectives, validate the findings and develop a comprehensive understanding of the processes and identified CMO configurations.

## 3. Results

**Figure 1.**
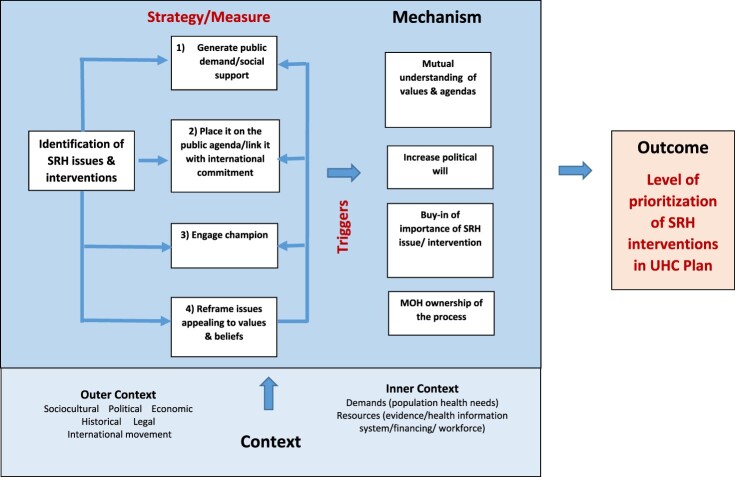
Analysis framework – what works, for whom, in what mechanisms, to what extent, in what contexts – to prioritising SRH services in UHC plans

In this section, we first present findings relating to the level of prioritisation of the three tracer interventions, based on the criteria laid out above (see Table 2). Next, we summarise the key strategies that Malaysian stakeholders adopted to promote greater prioritisation of these SRH interventions in the health system and UHC processes (Figure 1). Finally, we lay out the identified CMO configurations for each tracer intervention, and describe which contextual factors interacted with which mechanisms and triggers to generate the different levels of prioritisation.

**Table 2.** Outcomes: level of prioritisation of the three SRH tracer interventions

Health problem/issue	Maternal mortality	GBV	Deaths and complications due to unsafe abortion
	High MMR has been recognised as a significant population health issue in the 1960s – reduction in MMR is critical to improve overall population health	Recognised the physical and psychological harm suffered by victims of GBV **Not being perceived as a population health problem**	Maternal death due to unsafe abortion has not been acknowledged as a population health issue due to a lack of data and low MMR since 1990s
Health solution/ intervention	**Pregnancy and safe delivery care**	**One Stop Crisis Centre (OSCC)**	**Safe abortion and post-abortion care**
	Pregnancy and safe delivery care delivered through rural health services (RHS), supported by trained allied health personnel, have been seen as a cost-effective solution to reduce MMR from the 1960s to 1990s.	OSCC pilot – represented a feasible and successful model to respond to GBV through a multi-disciplinary, multi-sectoral and integrated approach using existing resources within the hospital, at no additional cost; has been perceived as a cost-effective solution. Special budget has been allocated to build the room/space for OSCCs but not the operational budget.	Safe abortion services – only recognised as a health solution, when pregnancy threatened the life, physical, and mental health of the mother No additional resources being allocated for abortion services in public hospitals – respective hospitals are expected to absorb the cost of the services within the O&G department
Level of prioritisation	**High**	**Medium/partial**	**Low**
	**Pregnancy and safe delivery care** are categorised as high priority services as MMR due to the risk of pregnancy and delivery has been perceived as a significant health problem and resources have been constantly allocated through Malaysia Plans and annual budget for such services	**One Stop Crisis Centres:** GBV has not been seen as a population health problem and only minimum budget is being allocated to ensure the space for OSCCs is available in the A&E department in all MOH’s hospitals.	**Safe abortion and post-abortion care services:** maternal deaths and complications due to unsafe abortion have not been seen as a population health problem, and there are no specific resources being allocated to support these services.
Coverage and financing scheme	**Public healthcare system**		
	All Malaysian women, regardless of age and marital status (paid or subsidised by the Malaysian government through taxation system) - Young people and unmarried women may encounter challenges due to stigma and discrimination - Parental consent is required if the woman is <18 years old - Indigenous women and populations of East Malaysia have difficulties in access due to the lack of knowledge and geographical barriers Non-citizen (migrant workers) – foreigner rate Refugee with UNHCR card – 50% discount of the foreigner rate	All Malaysian (paid or subsidised by the Malaysian government through taxation system) - Unmarried women and young people will face barriers because of social taboos around premarital sexuality and pregnancy Non-citizen (migrant workers) – foreigner rate Refugee with UNHCR card – 50% discount of the foreigner rate LGBTQI, undocumented migrant workers and refugees encounter greater challenges in accessing such services due to stigma and discrimination, cost and legal barriers.	All Malaysian women (paid or subsidised by the Malaysian government through taxation system) – under the circumstances when the mother’s life, physical and mental health is threatened - Written consent should be from the women herself. However, for Muslim couples, consent from the husband is also necessary - Parental consent is required if the woman is <18 years old Non-citizen (migrant workers) – foreigner rate Refugee with UNHCR card – 50% discount of the foreigner rate
	**Private healthcare system**		
	All Malaysians and Non-Malaysians can be covered – Mainly OOPE, cost is not being regulated and can be a barrier for lower socio-economic groups.	•	All Malaysians and non-Malaysians – Mainly OOPE, cost is not being regulated and can be a barrier for lower socio-economic groups.

### 3.1. Outcomes: level of prioritisation of the three SRH tracer interventions

#### 3.1.1. Pregnancy, safe delivery and post-natal care

Maternal health has been a national priority, and the focus of the public healthcare system since independence. Pregnancy and safe delivery services have been highly prioritised because maternal mortality due to the risk of pregnancy and delivery is deemed a significant population health problem, and maternal care and safe delivery services are viewed as cost-effective solutions (see Table 2). Resources including financial resources have been consistently allocated by the Malaysian government to strengthen the health system for these services since the 1960s. Both proportion of births attended by skilled health personnel and antenatal coverage for the first visit reached above 90% over the past 20 years.^29^

Free or almost free maternal health services are provided by the MOH's primary healthcare clinics and supported by secondary and tertiary care (the district hospitals and first-line referral facilities). Under the Fees Act, 1951 – Fees (Medical) Order 1982 (Amendment 2017), Malaysian women receiving antenatal and post-natal care at the MOH's primary healthcare clinics are only required to pay a negligible registration fee of MYR 1 (USD 0.25), while minimal fees are charged for delivery services at hospitals, ranging from MYR 10 to 1,200 (USD 2.5 to 300), depending on the ward classes and delivery procedures.^30^

“Government hospital, as a midwife I worked in a government hospital for 2 years and even go to the house and do delivery at home. So, everything is free of charge, even they develop complications, even the baby born, then go for immunisation, everything is free, from the time the Malaysian woman is confirmed pregnant.” *(IV16, Insurance provider)*

This high level of prioritisation of pregnancy and safe delivery services is mainly for married Malaysian women, and does not extend to unmarried women, adolescent girls and young women, and indigenous women. There is limited attention to addressing the different socio-cultural, religious, legal, financial and physical barriers that these women face in accessing these services.^31,32^ For young people, insufficient information, taboos, stigma and discrimination, and the legal requirement of guardian consent to access medical services constrain their access to early pregnancy care.^31^ This has contributed to a rise in adolescent pregnancies.^29^ In addition, the uneven distribution of facilities, personnel, medicines and equipment across geographies, is reflected in the comparatively low maternal health status of indigenous women and populations in East Malaysia, for whom late antenatal booking and home delivery have been common due to knowledge, language and geographical barriers.^21,29,33^

“So, you can say that number one, would still be geographical location that's a real barrier. There are a lot of communities living in Sabah and Sarawak, the nearest health clinic is days away … By walking … you cannot access by car, so many times they either have to walk through hills to somewhere where they can use a car to go to that facility … The mobile services, will fly in once a month and in bad weather, every 3 months.”

“MOH primary health clinics do have [youth friendly services]. How do we ensure that young people go there confidently, without stigma and discrimination and ensure privacy and confidentiality, we do not know.” *(IV1, NGO representative)*

In addition, the high prioritisation of access to maternal care also does not extend to non-Malaysians. In 2014, it was announced that the medical fees for non-Malaysians, including migrant workers, would be increased.^34,35^ They would be required to pay the full cost of medical fees at public healthcare settings in 2017, while refugees with the UNHCR card could receive a 50% discount off the full payment. The fee hike has become a significant barrier for many migrants and refugees to access pregnancy care and safe delivery services.^36^ In addition to financial barriers, undocumented migrants and refugees also face risks of security alerts and apprehension. Thus, many may forgo pregnancy care and safe delivery services and choose to deliver at home, which puts them at greater risk.^36^

“The foreigner rates have increased over the past few years, so that's quite a cost for them. … If they need to go to the hospital for delivery, they might have to come up with a deposit of RM 1400 (USD350) and the foreigners’ rate is RM 2800 (USD 700). With UNHCR card they get 50% of foreigner rates in the public hospitals and clinics. The coping mechanism is they might borrow from their communities, so many of the refugees are left with a lot of debt or they don’t get services at all, they wait until it's too late, complications set in.” *(IV 5, NGO representative)*

In addition to the public healthcare system, pregnancy, safe delivery and post-natal care services are also provided and can be accessed at private healthcare clinics or hospitals through OOPE. The antenatal and delivery costs vary between private healthcare clinics and hospitals in different states, as do the type of procedures.^37^ These costs are not normally being covered by private health insurance in Malaysia, which is risk-rated. Private health insurance companies in Malaysia are regulated by the Central Bank, which has adopted a risk-based capital framework, meaning that different insurers are allowed to set different premium charges, based on the risk profile for diseases of the individual.^38^ As pregnancy has not been perceived as a disease, the services have not been included in hospitalisation and medical benefits of the health benefit insurance package in Malaysia. As such, the high OOPE could cause households to incur catastrophic expenditures, especially non-Malaysians such as migrant workers and refugees who had difficulties in accessing public healthcare services, which might push them further into financial hardship.

“At the moment the market price in a private hospital for normal delivery in Kuala Lumpur is RM 7-8000 and if it comes to selective C-Section it comes to about RM 12-20,000, now RM 15-20, 000 I would say. Caesarean is not covered by private insurance. Pregnancy and delivery is not a disease, it's not a sickness.” *(IV16, Insurance provider)*

#### 3.1.2. GBV and OSCC services

Gender-based violence has received less attention in Malaysia. Since the enactment of the Domestic Violence Act in 1994, the Ministry of Women, Family and Community Development (MWFCD) is responsible for the national response to GBV, ranging from improving women's status and awareness raising on GBV to protection.^39^ However, GBV is still a “private” and “shameful” issue that is not to be discussed openly.^40^ It was estimated that 8% of women aged 18–50 years have experienced Intimate Partner Violence (IPV) in their lifetime;^41^ the figure may be an underestimate, given the sensitivity of GBV. Both preventive and protective services for GBV are scarce and there is no integrated national GBV policy framework for addressing GBV. At the same time, while the MOH is expecting to address the health impacts and consequences of GBV, GBV has never been perceived as a population health problem.

Nonetheless, One-Stop Crisis Centres (OSCCs) were introduced and are perceived as a potential solution without significant additional costs to the health budget, and these services have been integrated into the emergency and trauma departments (ETDs) in 129 MOH hospitals since 1996.^42^ Unlike pregnancy and delivery services, OSCC is only considered to be partially prioritised, as there is no extra budget allocation for this intervention (Table 2). The OSCC operation mainly relies on the existing EDT budget. It was reported that non-specialised hospitals at the district level struggled with a scarcity of resources, in particular, the lack of specialised staff and limited referral options.^43^ The utilisation rate of OSCC services remained unclear, due to the lack of official published national data. Although service data is available as an internal record, it does not feed back and translate into advocacy for the prevention of the health problem nor the allocation of a budget for the solution.

It was suggested that the main barrier for GBV survivors accessing these services was the low public awareness of the OSCCs, due to the lack of awareness-raising activities and the lack of trained staff and coordination. Furthermore, vulnerable and marginalised groups, such as LGBTQI, migrants and refugees, who were at greater risk of GBV, encountered even more challenges in accessing OSCC services, due to socio-cultural stigma and legal barriers.

“When I go to give lectures for the Women's Aid Organisation, they say ‘Doctor why don’t I see any advertisement on television about One-Stop Crisis Centre?” *(IV12, health provider)*

“So, the challenge we have with OSCCs is the fact that many doctors are not aware that putting the priority of the survivor first they insist on a police report before they are examined and this denies medical assistance and support for the survivor in many cases.” *(IV 5, NGO representative)*

“Rohingya communities quite prevalent among them to have high levels of domestic violence. The problem is that they are not documented that is a big issue, to make a police report and get the services.” *(IV 5, NGO representative)*

“This is the thing. Because we are still been seen as a man, so how can we be raped? Most of the time, transwomen wouldn’t want to go there [OSCC]. Not because they are naïve or they don’t care about themself. ‘Are they having anal sex? Are they sex workers themselves?' You put them in a situation where they will be looked into. Then are you selling yourself? Is this what your customer had done to you or something.” *(IV14, NGO representative)*

#### 3.1.3. Safe abortion services and post-abortion care

Safe abortion is categorised as having achieved a low prioritisation level with the UHC processes, because unwanted pregnancy is not deemed as a health problem in itself. As regulated by the Penal Code, abortion is only considered legal when the mother's life, physical and mental health is threatened (Table 2).^44^ While abortion is legally permitted under these circumstances, official published data on abortion are difficult to obtain, including the number of unsafe abortions in Malaysia. It was estimated that a total of 14% of total pregnancies ended in abortion in Peninsular Malaysia.^45^ Safe abortion services are available at both MOH hospitals with a gynaecologist's (specialist) support and in private clinics and hospitals. Although Malaysian women can access abortion services with a minimum fee at the MOH hospitals, the services were mainly performed surgically for medical reasons and hardly based on a woman's request.

“We have to provide what's spelt out in the law and the law is ‘only when the mother's health is at risk’. It's not like, you were naughty and you got pregnant and then you decide you want to terminate, we are not going to do, we are not allowed by law to do that.” *(IV7, current health policymaker)*

Medical abortion or abortion pills, such as misoprostol and mifepristone, are not legally available in Malaysia. Mifepristone has never been registered in Malaysia, and as for misoprostol, the drug company decided to cancel its registration and withdrew it from the Malaysian market, due to the complaints received about the misuse of the product.

“But what happened was we had two mothers died because of uterine rupture when they used misoprostol for induction of labour. Then what happened was, we had a few cases of people who got misoprostol and they decided to take it alone at home and they came into hospital on the verge of death. Because there was so much misuse of this drug … every time there's a complaint they go and complain to the drug company because they are supplying the drug. So to the extent that they just couldn’t handle it; they don’t want to be responsible anymore. So, the drug company went to the Ministry of Health and said that they wanted to withdraw the drug.” *(IV7, current health policymaker)*

Due to the limited access to medical abortion or surgical abortion at public healthcare facilities, private healthcare practitioners have filled part of the gap. However, they are generally discreet and the fees for such services remain unclear and they are not being regulated. Besides, access to abortion services in private settings is also mainly through OOPE and not covered by private insurance. The “exorbitant” prices potentially charged by private healthcare practitioners can make abortion inaccessible to the population from a lower economic status.

“We are certainly leaving it to the private sector, there may be exploitation. Those who do not know where to get an abortion and they can be charged exorbitant fees simply because they don’t know where to go.” *(IV3, NGO representative)*

“They will go to the private sector and some private doctors are unscrupulous and there are incidences where desperate women have been exploited and have been charged as high as RM 8000, for an abortion that normally costs less than RM 1000.” *(IV1, NGO representative)*

### 3.2. Mechanisms, triggers and context

#### 3.2.1. Pregnancy, safe delivery and post-natal care

Maternal health services, in particular pregnancy, safe delivery, and post-natal care have always been set as the country priority, specifically as the pillars of primary health care (PHC) (O). From the socio-cultural point of view, pregnancy within marriage was perceived as a “blessing and gift from God”, and this service was essential for married women who were in a “legitimate” relationship, because it involved “two lives at stake” (C) (Table 3). There has been strong political commitment towards these services since the early 1960s, driven by the high MMR as well as a need to cater to the predominantly rural population,^16^ who were the majority voters (C). The reduction of maternal mortality was perceived as the cornerstone of improving overall population health and national development.^16^

“For us, women have always been a priority in terms of access to these services for many it's 2 lives at stake. The morbidity and mortality involved are much more.” *(IV5, NGO representative)*

“Reproductive health, very important, it is considered, given a high-priority in the country … So yes, in terms of producing a healthy child … I think we have done very well.” *(IV9, former health policymaker)*

“In Malaysia plans the term used refers to improving the health of the rural population … It was the political system and because the rural population was ethnic Malay and they had the political power because they had the majority ruling party was elected by them. So, the democratic process which led to their being the majority, the ruling party had to provide for their constituency and so because it so coincided that the disadvantaged were the politically most powerful.” *(IV9, former health policy maker)*

Although the provision of maternal care within the marital context was not controversial, several key strategies were still employed in the 1960s to generate social support from rural communities, who were key stakeholders and normally delivered at home, due to traditional socio-cultural beliefs (C).

**Table 3.** Context-mechanism-outcome configuration

SRH Tracers	Context	Strategies/ Measures	Mechanism Triggered	Outcome
a) Pregnancy, safe delivery and post-natal care	**General context** Sociocultural acceptance of pregnancy within marriage; legitimacy due to two lives at stake **1st phase (1960s–1990s)*Outer context*** High MMR High levels of poverty 70% of the population resided in rural areas – - Rural population health needs and demands - Difficulties in access to care Local beliefs and customs on traditional medicines and home deliveries – valued the social and spiritual support received from traditional birth attendants (TBAs) during pregnancy and post-natal care ***Inner context*** Limited trained medical personnel, especially doctors	**Strategy 1: Generate public demand/ social support** through community participation and mobilisation – working with village development committees, NGO such FRHAM and National Family Planning Board (now known as National Population and Family Development Board) **Strategy 3: Engage champion** – trained midwives to work with TBAs	Strong demand and buy-in generated from the communities Increased political commitment since 1960s MOH ownership of the process	Increased the political will to invest and make pregnancy, childbirth and post-natal care as country’s health priorities. Budget and resources were committed and allocated in various country development plans to strengthen the health system to deliver these services. Significant reduction of MMR.
*** ***	**2nd phase (1990s – present)*Outer context*** Significant reduction of MMR in the 1990s – disease burden has shifted to non-communicable diseases Stagnant MMR since then until present Teenage pregnancy and stagnant adolescent fertility rate ***Inner context*** Stigma and discrimination from healthcare providers towards teenage pregnancies and unmarried women who access pregnancy and delivery care services	**Strategy 2: Link it with international commitments** – to achieve MDGs and SDGs **Strategy 4: Reframe issues appealing to values & beliefs** – Reframe the needs to provide pregnancy and delivery care for adolescents, young people and unmarried women under the umbrella of “Family Health”	Increased political will to provide continuing support for pregnancy and delivery care Created mutual understanding and buy-in on the need to provide pregnancy and delivery care for adolescents, young people and unmarried within healthcare providers and the community.	Pregnancy, childbirth and post-natal care have continued to be the country’s priorities with constant operational budget being allocated.
b) GBV and OSCC services	**From pilot intervention to scale-up*Outer context*** Perceived as a “private matter” that prevented others from intervening and prohibited victims from reporting GBV due to stigma, fear of retribution and socio-cultural beliefs. Burgeoning international movement on the rights of women and sexual and reproductive health in the early 1990s Domestic Violence Act passed under Penal Code in 1994	**Strategy 2: Link it with international commitments –** ICPD-PoA, Beijing Declaration and CEDAW **Strategy 3: Engage champion –** NGO working with health experts, engaging with champion from health sectors **Strategy 4: Reframe issues appealing to values & beliefs** – Reframe GBV as a health issue instead of rights issue	DVA enactment created legitimacy to engage health sector champion to generate buy-in of GBV solution (OSCC) Reframed the impact of GBV as a health issue to create mutual understanding and facilitate the buy-in The success of pilot OSCCs also increased the buy-in of MOH	OSCC has been partially prioritised as a feasible solution to address the health impact of GBV without additional operational budget support – mainly depended on the on the capacity of the hospitals as well as the volition of the decision-makers
c) Safe abortion services and post-abortion care	**Development of Termination of Pregnancy (TOP) Guideline (2012)*Outer context*** Perceived as a sensitivity issue – sociocultural norm deem abortion as taking a life as human life Legitimised and regulated by Penal Code **Inner context** Lack of data on maternal death due to unsafe abortion Lack of data on abortion services Health providers have vague interpretation of the legal context of abortion	**Strategy 1: Generate public demand/ social support** for the guideline: - including religious views and perspectives - introducing details on the eligibility and procedures for abortion, e.g. who can access, where and how abortion shall be conducted **Strategy 4: Reframe issues appealing to values & beliefs** – Reframe abortion services so as to address unsafe abortion and reduce MMR	Created the mutual understanding and buy-in at the top-management/policy making level	TOP guideline has been issued to all MOH’s hospitals. However, the services have not been prioritised. Services only available when there is a serious threat of medical complications. No specific budget being allocated for abortion services and the training for abortion services Healthcare providers may still not be comfortable to provide such services due to personal values or the lack of skills.

“We doctors, we go down and persuading families, we have mothers with pre-eclampsia and don’t want to go to the hospital and we are very concerned about maternal death, we could go down and persuade the husbands. Sometimes the husbands are waiting for us with apparatus, or whatever you call it, just to tell us ‘no don’t touch my family’. This is 40 years ago, traditional beliefs … ages ago that's how things were. I know then what the community likes. For instance, I conducted delivery at home.” *(IV6, former health policy maker)*

The first strategy was community participation and mobilisation^16^ to gain community support for ante- and post-natal care and safe deliveries and make individuals responsible for their health through the “village development committees” (M). In addition, the MOH also worked closely with NGOs, such as the Federation of Family Planning Association [now known as Federation of Reproductive Health Associations, Malaysia (FRHAM)] and the National Family Planning Board [now known as National Population and Family Development Board (NPFDB) under the Ministry of Women, Family and Community development] to increase the uptake of ante- and postnatal care, safe deliveries and family planning services, under the name of “improving families health” (M).

The second strategy was to partner with traditional birth attendants (TBAs), as the primary and most influential healthcare providers in the 1960s, especially in rural Malay communities.^14^ To increase the buy-in for safe deliveries by skilled birth attendants, the MOH engaged TBAs as champions to encourage women to utilise midwife clinics and health centres for antenatal and post-natal care and trained them to support the health clinics midwives during home deliveries (M).

The legitimacy of the services, strong demand generated from communities, and the commitment to reduce MMR increased the political will to invest and make maternal care a national health priority (O). Budgets and resources were allocated in various national development plans, including the five-year Malaysia Plan since 1965, to increase the number of healthcare facilities, expand the health workforce and strengthen the capacity of healthcare providers to deliver these services (O). As a result, the number of public PHC facilities and deliveries attended by skilled health personnel increased tremendously^16^. The rapid expansion of mother and child health and family planning services through the collaboration with FRHAM and NPFDB, as well as the expansion of PHC clinics in rural areas, were major factors in lowering infant and maternal mortality rates across the country from 1960 to 1990.^46^

“While we are doing this, we have a lot of resource support, nurses have been trained, we have public health nurses … they are the ones that manage the clinic actually … they know the SOPs, where you are doing, how you are doing, very connected with the community … ” (IV6, former health policy maker)

The prioritisation of maternal care for all married Malaysian women played a key role in the significant reduction in MMR, but MMR has remained relatively stagnant over the last 20 years (C).^29^ Teenage pregnancy remains a concern^29^ and adolescents and unmarried women often face barriers, such as stigma and discrimination in accessing these services due to socio-cultural and religious norms and practices (C). Several strategies have been adopted to justify the continued prioritisation of maternal care, particularly to address the needs of unmarried and young women. The first strategy was to link it with international commitments, such as the Millennium Development Goals (MDGs) and the SDGs (M), namely, to promote the achievement of the MDG target of 11 per 100,000 live births. The second strategy has involved reframing it under the umbrella of “Family Health”, which emphasised a life course approach to address the needs of all people, including unmarried women or young people (M).

“We won’t label SRH in UHC, but we always label it the other way around, Family Health. SRH is subsumed under family health since 90s and what is family health? Family health is the life-course approach and if it happens to be adolescents, it's adolescent health, if it happens to be a woman, it's women's health and if happens to be a man, it's men's health. So that's how we integrate it. Whatever context you want to discuss in SRH it's all in family health and I don’t think they miss anything.” *(IV6, former health policymaker)*

These strategies provided a strong justification for the continuing buy-in and support from policy makers and pregnancy, childbirth and post-natal care services remained as one of the country's health priorities with a constant financial allocation throughout the years.

“The operational budget for MCH won’t be cut, but if the budget is insufficient, they will cut the training budget or the budget to build a new facility first.” *(IV 20, current health policymaker)*

#### 3.2.2. GBV and OSCC services

While the Malaysian government and MOH recognised that there was a need to address the increasing number of domestic violence, rape and sexual abuse cases referred to government since the enactment of the Domestic Violence Act (DVA), gender-based violence (GBV) had never been seen as a population health problem that needed to be highly prioritised (O) (Table 3). One of the reasons for GBV being less prioritised could be the under-reporting that is linked to stigma, fear of retribution and socio-cultural beliefs (C). At most times, GBV has been perceived as a “private matter”,^40^ and this precluded outsiders from intervening and prohibited victims from reporting (C).

“In terms of community and society we are very patriarchal and there's this belief, especially amongst Muslims that they have a right to beat their wives. Of course, it's not literally beating their wife.” *(IV2, NGO representative)*

GBV had not been addressed until the Domestic Violence Act (DVA) was passed under the Penal Law in 1994 (C), which subsequently led to the establishment of the One-Stop Crisis Centers (OSCC). Several key events had created a window of opportunity for NGOs to work with health experts to change existing policies. First, there was a burgeoning international movement on the rights of women and sexual and reproductive health, including the 1994 International Conference on Population and Development (ICPD), Beijing Declaration and Convention on the Elimination of all Forms of Discrimination Against Women (CEDAW). The international movement gradually influenced views within the country, and NGOs comprising SRH advocacy groups and women's groups strongly advocated for GBV to be prioritised as a national agenda. The advocacy efforts were fully supported by a prominent individual political champion – Napsiah Omar, the late National Unity and Social Development Minister, who had been active on women's issues and led the Malaysian Government Delegation in the negotiation process to finalise the ICPD-PoA,^47,48^ which led to the enactment of the Domestic Violence Act (M).

“The first Beijing conference brought up 11 factors/11 issues and health were one of the issues … and from health, reproductive health was one of the key issues talked about, then you have CEDAW and this declaration of the year against violence against women, those are the key factors.” *(IV2, NGO representative)*

Secondly, the DVA enactment created legitimacy for NGOs to engage champions from the health sector to advocate for the need to assist survivors of GBV and address the impacts of GBV (M). These advocates and NGOs also reframed GBV as a health issue, which was more acceptable and less sensitive than a “rights” issue, and facilitated buy-in from a broader range of stakeholders (M). Shortly after, a pilot programme of the OSCC was undertaken by the head of the ETD in Hospital Kuala Lumpur. All services including medical, counselling and police services were provided under the ETD department, while legal aid and religious support were provided upon referral.^43^ Clinical services were mainly delivered by the hospital, while counselling services were provided by volunteers from women's NGOs. With the close working relationship with key stakeholders including the police, social welfare department, legal aid and NGOs, and with multidisciplinary support within the hospital, the OSCC pilot represented a feasible and successful model to respond to GBV using existing resources within the hospital, at no additional cost to the MOH.^43^ This eventually gained their high-level support (M).

“Without the Domestic Violent Act, you would not have OSCC for sure.” *(IV2, NGO representative)*

“Dr Abu Hassan is the one who championed the OSCC, not only in Hospital Kuala Lumpur but took it out of the country … I think it helped because of his position, he is the head of trauma and his position gives him the clout to speak about the issues … . The Domestic Violence Act is a good Act which needs this support and this intervention, it gave him a good platform to work on.” *(IV2, NGO representative)*

“Health is an easier entry point; people don’t see it as a clash or a confrontation. Because when you talk about women's health in a patriarchal society people don’t believe women should have that much rights, they are very confrontational. They are very defensive. But when you look more from a health perspective, people can recognise and see it as a health issue and it's also good to bring awareness.” *(IV2, NGO representative)*

In 1996, the MOH directed all MOH hospitals to integrate OSCCs within their ETDs^49^ (O). However, the services were only partially prioritised, since no additional budget or resources were allocated to support the OSCC services (O). Even though an OSCC policy and guidelines subsequently issued in 2015 stated that “*MOH shall be responsible for the availability of standard infrastructure at all Emergency and Trauma Departments”* and special provisions would be allocated to build the room or space for OSCCs,^49^ the ETDs were still expected to absorb the operational and training costs for OSCCs.

“There's no special budget, there's no special budget. We make do with the resources we have. The consumables are available, the staffing.” *(IV12, health provider)*

“We have clinical practice guidelines, all government hospitals must allocate, or endeavour to allocate in their future budgeting a specialised room to manage these survivors.” *(IV12, health provider)*

Due to the lack of financial allocation, the prioritisation and actual implementation of OSCCs have mainly depended on the capacity of the hospitals as well as the volition of the decision-makers, in particular the Head of ETD at the respective hospital (O). Non-specialised hospitals at the district level have struggled to cater to the needs of GBV survivors, due to the lack of budget, specialised staff and insufficient training.^21,43^

“There's no budget [training]. We organise and maybe get our doctors and nurses to pay a fee, for the food.” *(IV12, health provider)*

“Maybe one [challenge] is if it's a small district hospital is the logistics; you have a rape survivor coming to a district hospital. There are no specialists, there are no OBGYN specialists, so you have to send this patient via transport, hospital transport to a tertiary centre with an obstetrics and gynaecology specialist.” *(IV12, health provider)*

#### 3.2.3. Safe abortion services and post-abortion care

As shown in Table 3, safe abortion and post-abortion care were the least prioritised (O) within national UHC processes, which is primarily due to the cultural sensitivity of the issue (C). Maternal death due to unsafe abortion has not been seen as a population health issue, given the lack of data on unsafe abortions and post-abortion care and low MMR in Malaysia (C).

“Abortion has always been a taboo subject. I’m not sure we should talk about abortion because at the moment everything is going well, in that under the counter, private/public sector … You put it on the table, you are attracting attention to it, so I for one say let sleeping dogs lie.” *(IV9, former health policymaker)*

“I don’t think you can get abortion data easily; we are not in a community where people will disclose this easily … They will never disclose and I don’t think it's peculiar for Malaysia because abortion in any family, whether it's Western, Asian or Middle-Eastern, will take it very differently, it's not something like eating food. It's very sensitive.” *(IV6, former health policymaker)*

Abortion services have only been recognised as a need or a health problem that needs to be addressed when it threatens the mother's life, physical or mental health. The majority of healthcare providers have not been clear about the legal provision of abortion services as the Penal Code regulates it (C).^50^ Due to the sensitivity of the issue and vague interpretation of the laws (C), most healthcare providers, particularly those in public healthcare settings, would not want to offer it.^50^ As for most of the private healthcare providers, they would keep a low profile in providing such services.

As such, the Obstetrics and Gynaecology (O&G) Committee under the MOH decided to issue the Guideline of Termination of Pregnancy (TOP) for all MOH hospitals in 2012 to provide clearer guidance. The health experts mainly led on the development of the TOP guideline. NGOs that had strongly advocated for accessible and affordable safe abortion services, such as Reproductive Rights Advocacy Alliance Malaysia (RRAAM) and the Federation of Reproductive Health Associations, Malaysia (FRHAM), were not involved or consulted. The guideline also included religious views and perspectives on abortion (e.g. conditions that allowed for abortion from religious perspectives) as appendices to increase the buy-in and support of healthcare providers, as well as policy-makers (M).

“Abortion services come under the penal code, it doesn’t come under the civil code. So, there was a lot of confusion among practitioners, whether they can do or whether they cannot do … The law says whenever maternal health is at risk you can do and then, of course, we had confusion as to whether we can offer to all religions, or only certain religious communities and not to the rest … So we decided that we need to have a guideline … then we fine-tune it, we took it to the Director-General of Health,  … who went through it line by line, because it was a sensitive document.”

(IV 7, current health policymaker)

To get the top decision-makers’ buy-in and create ownership within the MOH, the guideline had reframed abortion as a need to address unsafe abortion, as well as one of the strategies to further reduce MMR (M).^51^ The guideline also stated that termination of pregnancy should only be provided at hospitals at the tertiary level with specialist support and two doctors, one of whom had to be a specialist and would need to assess if an abortion is necessary.^51^ This approach was intended to generate support and create mutual understanding among healthcare providers, even though the law (Penal Code 312) states that only one medical practitioner's diagnosis is required to determine if the pregnancy may harm the mother's physical or mental health (M). While the specification of the criteria/condition in the TOP guideline aimed to generate buy-in among healthcare providers towards the provision of abortion services, the discrepancy in the prerequisite for abortion stated in the law and the guideline might have created confusion and barriers for patients to access the service, which could have led effectively to further deprioritisation or rationing of this SRH intervention.

“This policy is for Ministry of Health hospitals, to safeguard the reputation and registration of healthcare providers to a point that, although the Malaysian law says that consent must be signed by one registered medical practitioner. In this guideline, it's two … That's the reason, to protect, people are very sensitive, so we don’t want people to say ‘you alone made the wrong decision’.” *(IV7, current health policymaker)*

While the TOP guideline was approved by MOH and was disseminated to all MOH hospitals, it was unclear how far abortion services were prioritised at the hospital level (O). First, no specific budget was allocated for abortion care services and respective hospitals were expected to deliver these services under the operational cost of the O&G department (O). Second, some healthcare professionals might still not know or lack understanding of the guideline or not feel confident or comfortable performing the services (O). Third, the MOH only provided services where there was a serious threat of medical complications (O). As such, most people might still not be aware that the services had been integrated into the O&G department at public hospitals or find it very difficult to access, due to the restrictions and limitations.

“It's not like, you were naughty and you got pregnant and then you decide you want to terminate, we are not going to do, we are not allowed by law to do that. If you have an abnormal baby and the baby is going to die, the law does not give me the power to terminate that pregnancy. I only can terminate if the mother's health is at risk. And the other thing, let's say, you get raped and you get pregnant, I can’t terminate the pregnancy … only if I can say this person is on the verge of a mental breakdown … that in the confines of the law.” *(IV7, current health policymaker)*

Moreover, the guideline does not make it compulsory for hospitals to provide the service even if the patients meet the prerequisite (O). Medical practitioners could choose not to treat the patients and refer them to other hospitals. The socio-cultural norm that deems abortion as immoral affects decision-making and rationing by medical practitioners (C).^50^ Clearly, the guideline was not sufficient to promote and trigger the mechanisms of mutual understanding of values and agendas required for the effective prioritisation of safe abortion and the healthcare provider level (M).

“Somewhere in 2014/2015, we found some people did not even know this guideline is there, so then we re-started the process of sending it out. But some hospitals may not feel confident doing it, or comfortable to do it, so we don’t force anybody to do … refer her to the nearest hospital that can provide the service, government hospitals … these are government guidelines.” *(IV7, current health policymaker)*

## 4. Discussion

This study documents the extent of integration and prioritisation of three SRH interventions in Malaysia's national UHC plans and processes, including pregnancy, delivery and post-natal care services; GBV services; and safe abortion and post-abortion care. While Malaysia has made significant efforts and progress in providing free (and almost free) care for most SRH services (including contraception, antenatal, delivery and post-natal, safe abortion and post-abortion care, gender-based violence, STI and HIV prevention and management, cervical, breast and prostate cancer prevention, screening and treatment), at various levels of the public healthcare system for all Malaysians, the study provides evidence that the level of integration, coverage and prioritisation of different SRH services varies. The study used realist synthesis to provide insights into why and how specific services come to be prioritised, through the exploration of the contextual factors and the processes and pathways that can enable the integration of SRH services in UHC. The findings demonstrate the importance of looking at the interaction of strategies and approaches, mechanisms and context to understand the priority-setting process, particularly when seeking to integrate sensitive and controversial issues and services such as some SRH interventions.

One of the first steps to ensure they are integrated and accessed, is for these health issues to become political priorities and priority health policies. Some evidence on maternal mortality prioritisation suggests that MMR reduction, which has been perceived as a measure of the success of a country's development, provides an incentive for greater prioritisation of maternal care.^52^ Similarly, in Malaysia, the prioritisation of and investment in pregnancy, safe delivery and post-natal services have been perceived as the cornerstone of improving overall population health and national development,^16^ which triggers strong buy-in from policy-makers to set and maintain maternal care as a high priority with reliable budgetary resources.

While the strategies contributed significantly to reducing MMR in Malaysia, our study found that it had reached its maximum benefits. Malaysia was unable to achieve the MDG target of 11 per 100,000 live births (representing a reduction by three-quarters from 44 in 1991) in 2015^29^ and the MMR has remained at around 25–30 per 100,000 live births over the past 15 years. Even though pregnancy and delivery services remained the country's top priority, one of the reasons that the MMR could not be reduced further might be that the focus of the services only targeted married Malaysian women. Studies showed that marginalised populations, such as unmarried women, adolescents and young people, migrants and refugees, had encountered social and legal barriers in accessing these services.^21,31^ The Malaysia MDGs Report 2015 indicated an urgent need to address teenage pregnancies and unmarried women's and young people's access to SRH services.^29^ As indicated in the report, there were over “18,000 antenatal visits” by teenage mothers in 2011 and 2012.^29^ The adolescent fertility rate (AFR), which stood at 13.4 births per 1,000 women ages 15–19 in 2018,^53^ has not improved since 2000.^54^ Hindering factors for further reductions in MMR and AFR could be attributed to the lack and poor quality of comprehensive sexuality education (CSE) and contraceptive practice.^55,56^ Family planning has not been a national priority due to declining fertility rates, which is reflected in a Contraceptive Prevalence Rate (CPR) that has stagnated at just over 50% for all methods among married women of reproductive age since the 1980s.^57,58^ To further improve MMR, AFR and CPR, wider community support will be important through the involvement of key community gatekeepers, such as parents and religious leaders, to ensure that young and unmarried people, especially, access CSE and contraception.^59^

Second, the study found that the priority-setting process for several SRH tracer interventions was closely associated with international movements. As has been the case in other settings, these international movements played a key role in establishing a global norm about the unacceptability of maternal death, which subsequently influenced Malaysia to embrace the cause and prioritise pregnancy and safe delivery services.^13^ On the other hand, Malaysia's active involvement in the negotiation process for finalising the ICPD-PoA in 1994, and continued participation in ICPD-related activities in the 1990s, also provided impetus to the prioritisation and resource allocation for SRH programmes under the umbrella of “population, family development and women's development programmes” in the country.^47^ In the case of GBV, the international movements created momentum for the women's movement in the country. In contrast to Ravindran and Govender's findings on the involvement of civil society organisations and communities in priority-EPHS,^7^ a robust network of women-led NGOs was formed, with individual political champions’ support, and led to the passing of the Domestic Violence Act (DVA) in 1994, which subsequently provided legitimacy for the OSCC pilot.^43^ The country's subsequent commitment to international agendas, such as the MDGs and SDGs, also provided a justification for policy-makers to continue the financial allocation for pregnancy and delivery services, as well as to expand it for unmarried women and address unsafe abortion to further reduce the MMR. Although linking SRH issues with international commitments could facilitate prioritisation at the macro level and in national policy frameworks, this did not necessarily translate into prioritisation at the meso and micro level, where resources were limited. For example, the level of prioritisation and actual implementation of OSCC at hospitals at the district level was low, due to a lack of resources and support within the health system.^43^

Third, engaging champions was found to be another successful strategy to advocate for SRH tracer interventions to be prioritised. Champions have been defined as “*individual(s) who dedicate themselves to supporting, marketing and driving through an implementation, overcoming indifference or resistance that the intervention may provoke in an organization”.*^60^ Engaging champions, such as including an influential political leader, healthcare provider, informal leader, religious group, or prominent opinion leader or groups within the community, has proven to be a practical approach to promoting evidence-based practices and advance public health issues.^13,61^ A review article has demonstrated the importance of building alliances with sympathetic champions in government and civil society to translate research outcomes into SRH health policy making process, especially in countries with limited resources.^62^

Similar to Shiffman's study^13^ that found willingness of national political champions to be one of the key factors that generated political priority for maternal mortality reduction in developing countries,^13^ our study also showed that engaging the “insider” group, in particular, healthcare providers within MOH's policymaking circles, seems to enable greater success. The likelihood of policy change occurring with the advocacy of an “insider group” could be due to their influences and privilege in access to the authority and executive.^63^ Nonetheless, the expected outcomes could vary, depending on various factors, such as the structure of power and authority, the evolution of the champion's role and duties over time, partnership with local stakeholders, and the change in a contextual environment. For example, the success of the OSCC pilot and advocacy efforts by women's NGOs and influential hospital staff created buy-in by the MOH, which drew up a formal policy for OSCC to be scaled up in 1996. However, the influence of the NGOs and health providers could not be sustained and duplicated at the district level, due to the shift in the political concern for GBV and the lack of an internal successor to continue to champion the issue.^43^ As for abortion services, despite there being a champion to push for the development and dissemination of an abortion guideline at the policy level, partnerships were not established with local stakeholders, especially healthcare providers at public hospitals, and their buy-in towards the provision of abortion remained unclear.^63^ As such, continuous engagement with champions from different sectors and constituencies could be particularly important to institutionalise SRH services, especially for issues that require expertise and service delivery platforms beyond the health sector. For example, the high coverage and institutionalisation of the human papillomavirus vaccine in the school-based vaccination programme in Malaysia have demonstrated how inter-agency and multi-sectoral collaborations can contribute to sustained integration of SRH services.^64^

Lastly, “reframing” was identified as an alternative strategy adopted for priority-setting for sensitive SRH services, such as GBV and abortion, as well as providing justification for addressing the SRH needs of marginalised populations. Framing ideas on the problem and its potential solution in a particular way and taking the values and contexts into consideration, helped to engage stakeholders, mobilise specific policy responses and shift the terrain of the debate.^65^ A previous study has shown that both internal and external framing – generating consensus on framing the problem and its potential solution among internal (e.g. policymakers, health providers, etc.) and external (community members, religious leaders, etc.) stakeholders – was critical to gain political support for controversial issues such as SRH.^66^ Negative framing of sensitive SRH issues could result in significant stigmatisation and affect the level of prioritisation.^67^

In our study, while internal framing had demonstrated success in generating consensus on the importance and provision of OSCC and abortion services, both services did not seem to be included in other parts of the health planning process, including costing and budgeting, operational planning, and monitoring and evaluation mechanisms. This lack of integration in the health planning process and insufficient financial commitment could be due to the lack of external framing of the issues, which were influenced by socio-cultural norms and religious beliefs. The findings of our study were similar to a study on the prioritisation of adolescent SRH services in Kenya, where the divisions between internal and external framing of SRH services for adolescents led to the low prioritisation of such services.^66^ Reluctance to discuss and address these issues or restrictions to accessing certain SRH services for people who do not conform to socially accepted norms of behaviours has posed constraints and challenges in prioritising and integrating SRH services in UHC in most countries, including Malaysia.

The study has several limitations. First, the analysis focussed on the integration and prioritisation of the three SRH tracer interventions in the public healthcare system, rather than the health system as a whole. This limits our understanding of the role of private healthcare providers in universal access to these SRH services, including for non-Malaysians. Second, data collection and analysis were conducted within a short period (January to mid-March 2020) and affected by the COVID-19 outbreak. The research team had difficulties reaching key informants at the initial stage, especially current health policy-makers, as they were occupied with the COVID-19 response. Therefore, their perspectives are less represented in the findings of the study. However, several key informants, who were former policy-makers and had been involved in the priority-setting process, provided rich data and perspectives, in particular relating to the historical background, processes, and contexts, and how prioritisation decisions had been made. Moreover, interviews were conducted with stakeholders from various backgrounds and constituencies, including representatives from civil society and academicians, which provided different and complementary insights into past decisions and processes from within and outside government.

## 5. Conclusion

Priority-setting is as a key process for the integration of SRH in UHC. It is a political decision-making process that reflects societal values and norms to select which SRH issues and solutions should be prioritised, provided and invested in, based on certain criteria. Various strategies were adopted to advocate for greater prioritisation of SRH services in the public healthcare system in Malaysia. The less controversial SRH issues, such as pregnancy and delivery services, and those deemed within minimal additional costs to the health budget, such as OSCC for GBV survivors, can get buy-in from stakeholders. Strategies that were found to trigger this buy-in from internal stakeholders for priority-setting in the Malaysian context included: the generation of public demand and social support; placing SRH issues on the public agenda or linking them with international commitments; engaging with champions within government; and reframing SRH issues to appeal to existing values and beliefs. However, it was also observed that these strategies were influenced by the outer context, which led to different levels of prioritisation. The prioritisation of SRH in UHC would need to be pursued differently for the different types of SHR services, especially for sensitive services like GBV and safe abortion services, which require engagement with both technical enablers (criteria set and strategies) and contextual enablers (socio-cultural norm and values). As such, the integration of comprehensive SRH services in national health policies, strategies and plans for UHC will require identifying policy windows and enabling conditions to advance specific SRH interventions, and a continuous prioritisation or re-prioritisation to increase and sustain health system resources that are allocated to advance universal access, where no one is left behind.

## Footnotes

* “Comprehensive SRH services” defined by WHO-RHR – Including contraception, pregnancy, childbirth and maternal care (antenatal, delivery and postnatal), fertility care, safe abortion and abortion care, sexual function, gender-based violence, female genital mutilation, LGBTQI, menstrual care, STI and HIV prevention and management, cervical cancer prevention/screening/treatment, breast and prostate cancer screening/treatment.
